# Prevalence of Post-traumatic Stress Disorder in Adult Burn Patients: A Study From Urban Tertiary Care Hospitals in Pakistan

**DOI:** 10.7759/cureus.81969

**Published:** 2025-04-09

**Authors:** Muhammad Haris Khan, Abdul Haseeb, Maryam Nadeem, Gulan Zafar, Faiqa Hashmat, FNU Ariya, Sohaib Rashid, Noman Khan, Sonia Shakeel, Azhar Ud Din Babar, Muhammad Musawir Niaz, Muhammad Abdullah Khan, Laeba Jamil, Muhammad Sufyan Darwesh, Adeela Mustafa

**Affiliations:** 1 Medicine, Khyber Medical College, Peshawar, PAK; 2 Medicine, Ayub Medical College, Abbottabad, PAK; 3 Surgery, Khyber Medical College, Peshawar, PAK; 4 Community Medicine, Khyber Medical College, Peshawar, PAK

**Keywords:** adults, age groups, burn injuries, burn patients, gender, post-traumatic stress disorder, socioeconomic status

## Abstract

Introduction

Post-traumatic stress disorder (PTSD) is one of the main psychological conditions that develops after a traumatic event such as a burn injury. Patients often experience flashbacks, nightmares, anxiety attacks, and endless thoughts about the traumatic burn injury, which deteriorate their quality of life. These patients are usually socially isolated, have low self-esteem, and are more likely to commit suicide. Burn patients, who are particularly vulnerable, often do not receive adequate post-burn care, and psychological disorders like PTSD are often overlooked in developing nations such as Pakistan. Hence, this study investigated the prevalence of PTSD among adult burn patients in tertiary care hospitals and examined its distribution across different age groups, genders, and socioeconomic classes to highlight its overall burden as well as differences among these groups.

Materials and methods

This cross-sectional study was conducted from January 2024 to July 2024, recruiting 275 adult burn patients from tertiary care hospitals in Peshawar, Pakistan, using non-probability convenience sampling. Non-cooperative, unwilling, unconscious, and severe burn injury patients were excluded from the study. Additionally, individuals under the age of 18 years and those whose burn injuries occurred less than a month prior were omitted, as PTSD requires the traumatic event to have happened more than a month ago for diagnosis. All the burn patients were at different stages of recovery. A provisional diagnosis of PTSD was made by using the PTSD checklist for DSM-5, a 20-item checklist that assessed the symptoms of PTSD via a five-point Likert scale. The socioeconomic status was determined via a modified form of the Kuppuswamy Socioeconomic Status Scale.

Results

The diagnosis of PTSD was made in 52% (143 out of 275) of people who suffered burn injuries. The prevalence of PTSD in males and females was 40% (59 out of 148 males) and 66% (84 out of 127 females), respectively. Sixty-two percent of retirement-aged and 57% of middle-aged patients were mostly affected by PTSD. Similarly, PTSD was more prevalent in lower-class patients, i.e., 69% of patients with burn injuries from lower socioeconomic backgrounds were affected.

Conclusion

An alarmingly high prevalence of PTSD was found in burn patients. It was more prevalent in females than in males. A greater percentage of retirement-age and middle-aged adults were diagnosed with PTSD. When compared to patients from various socioeconomic backgrounds, burn patients from lower socioeconomic backgrounds were found to have a higher prevalence of PTSD.

## Introduction

A lot of traumatic events in life can adversely affect an individual’s health in every aspect. Burn injury is also one of those events. A burn survivor has to go through a lot of complex challenges in life. Starting from dealing with the actual burn injury, then the trauma of the burn accident, and finally dealing with the reintroduction into society [1]. These patients must address their changes as well as people’s reactions to those changes. They have to deal with the physical and psychological scars inflicted by the burn injury. These scars and disfigurements often lead to low self-esteem and social isolation. As a result of all these risk factors, burn survivors often demonstrate higher levels of suicidal tendencies [2]. Major burn injury is life-threatening and is associated with high morbidity and mortality rates [3]. Burns, whether deliberate or accidental, are the fourth most frequent type of trauma worldwide [4]. Burn injuries account for a large number of public health problems worldwide, often leading to a great number of psychosocial and psychological issues that may occur after the trauma of burn injuries [5]. Some common psychological issues that are of common occurrence after the trauma of burn injuries are depression, anxiety, and post-traumatic stress disorder (PTSD) [5]. There are also a large number of post-traumatic adaptations that can occur following a burn injury, such as dissociation, somatization, sleep disturbances, a history of substance abuse, and various other issues that may ensue after such a trauma [2]. PTSD is one of the most common disorders that may occur as a result of these traumatic injuries [6]. According to a study, 42% of people have PTSD six months following an injury [7]. Furthermore, research indicates that 23% of people who survive a serious injury a year after their initial hospitalization suffer from PTSD [8]. PTSD is a psychiatric disorder that can occur after experiencing a traumatic event such as a burn injury [9]. People with PTSD often have exaggerated reactions to anything that reminds them of the traumatic event [10]. Amazing improvements in burn survivors' care and treatment have significantly decreased mortality, which has increased the number of burn survivors in the post-traumatic adaptation phase [11].

Burn survivors not only need medical care for their wounds, but they are also in dire need of some special interventions that are focused on providing physical, social, and psychological care to these patients [12]. As part of their medical care, burn victims need wound care, fluid resuscitation, pain control, and infection prevention. PTSD and psychological discomfort can be addressed with behavioral therapies such as peer support groups, trauma-focused counseling, and cognitive-behavioral therapy (CBT). Plastic surgery, along with psychotherapeutic care, may help in the reintegration of these patients into society. A recent study done on young burn survivors in Taiwan concluded that young burn victims made a remarkable physical and psychological recovery under supportive care in two years [4]. Any post-burn mental health issues should be detected as early as possible, and necessary steps should be taken to avoid any undesirable consequences [4]. Nursing staff may play an important role in helping burn victims identify certain mental health issues and how to deal with these issues long-term [13]. One other intervention that shows promise in dealing with the trauma of the burn accident is peer support from fellow burn victims [14]. PTSD has debilitating effects on burn victims, which calls for its early intervention. Cognitive behavioral therapy (CBT) has shown great promise in preventing the crippling effects of PTSD; however, for many people it is of no use [9]. Reducing post-burn anxiety and depression is also an important aspect of the care of burn patients [15]. The support of family and friends may also help a burn patient get back on his feet. All necessary measures should be taken to ensure that burn victims regain their place in society with optimal physical and mental well-being, free from anxiety, depression, PTSD, or any other challenges.

Although the psychological and social aspects of post-burn care are given great importance in developed countries, no such consideration is given to it in developing countries like Pakistan [16]. In developed nations, comprehensive treatment approaches that emphasize holistic recovery include the psychological and social aspects of post-burn care. On the other hand, these factors frequently get less attention in developing countries like Pakistan. According to a study conducted in Pakistan, burn patients' PTSD symptoms were significantly predicted by their lack of social support, suggesting a deficiency in psychosocial care [17]. Additionally, studies highlight the scarcity of standardized, easily available, and long-lasting psychosocial therapies for burn survivors when they move from the hospital to their homes [18]. These results point to the urgent need for developing nations' post-burn care practices to provide psychosocial assistance. A recent extensive study done across four low and middle-income countries, i.e., Nepal, Rwanda, Sierra Leone, and Uganda, found that low and middle-income countries were responsible for 90% of burns, and no importance was given to the proper treatment of burns in such countries [19]. The cases of accidental burns (51%), stove burns (34%), acid burns (13%), and burns related to domestic disputes have increased at an alarming rate in Pakistan in recent years [20]. PTSD and other mental health issues are under-recognized and under-treated in a developing nation such as Pakistan. Although this important issue has been widely researched worldwide as well as in certain cities of Pakistan, no such research has been conducted in Peshawar, which has an overburdened health system, and there is less emphasis on fourth-tier rehabilitation programs. It is crucial to understand how post-traumatic distress and adaptation manifest themselves in burn survivors in a social and economic environment as underdeveloped as Peshawar. This study will shed light on the mental health issues that may ensue after a traumatic burn injury and the necessary interventions needed for the rehabilitation of burn patients and their families. It will also highlight the importance of post-burn care, especially psychological and psychosocial care. Also, this study will familiarize health policymakers with the difficulties burn survivors encounter during their recovery phase and can help them formulate measures to improve their quality of life, particularly concerning their psychosocial adaptation. Hence, this study explored the prevalence of PTSD among adult burn patients in tertiary care hospitals, analyzing its distribution across various age groups, genders, and socioeconomic classes to emphasize both its overall impact and the disparities among these groups.

## Materials and methods

Study design and setting

This cross-sectional study was conducted from January 2024 to July 2024 in tertiary care hospitals of Peshawar, Pakistan. The study adhered to the Strengthening the Reporting of Observational Studies in Epidemiology (STROBE) checklist to ensure methodological rigor. Data were collected from the following hospitals: (1) Burns and Plastic Surgery Centre, Peshawar; (2) Khyber Teaching Hospital (KTH), Peshawar; and (3) Lady Reading Hospital (LRH), Peshawar.

Participant characteristics and sampling methods

The study included 275 adult burn patients (aged 18 years or above) who were either admitted or were patients of the outpatient departments (OPDs) at the selected hospitals. Inclusion criteria required patients to have experienced PTSD symptoms for more than a month, as PTSD is diagnosed at least one month after the traumatic event. Non-cooperative or unwilling, unconscious patients, and patients with severe burn injuries causing extreme pain, preventing their participation, were excluded from the study. We used non-probability convenience sampling for participant recruitment, and the sample size was determined using Cochran’s formula.

$n = \frac{Z^2 \cdot p \cdot (1-p)}{e^2}$

Here, Z = 1.96 (for 95% confidence level), p = 0.233 (23.3% prevalence from a previous study), e = 0.05 (margin of error). Substituting these values into the sample size formula as follows.

$n = \frac{1.96^2 \times 0.233 \times (1 - 0.233)}{0.05^2}$

$n = \frac{3.8416 \times 0.233 \times 0.767}{0.0025}$

$n = \frac{0.6887}{0.0025}$

$n = 275$

Thus, the required sample size for this study was calculated to be 275 patients.

Questionnaire

The questionnaire had three sections (appendix 1). The first part had questions related to demographics. According to the UN statistical division of age groups, the age groups described in Table 1 were included in our study.

**Table 1:** UN statistical division of age groups.

Age groups	Age range (years)
Young adults	18-24
Middle-aged adults	24-44
Older adults	45-64
Retirement age	65 and above

The second part had queries related to the patient's socioeconomic status, which was used to categorize them into upper, upper middle, lower middle, upper lower, and lower classes, respectively, via a modified form of the Kuppuswamy Socioeconomic Status Scale with values ranging from below 5 to 29 (appendix 2). The third part of the questionnaire consisted of the PTSD checklist for DSM-5 (PCL-5), which was used to assess the symptoms of PTSD the patient had experienced in the past month. A cut-point score of 31-33 was considered positive for PTSD out of a total score of 80. It was a 20-item checklist and assessed symptoms of PTSD via a five-point Likert scale ranging from 0 (not at all) to 4 (extremely) (appendix 3). The Cronbach’s alpha value for all the items of PCL-5 was 0.94 [21].

Procedure

Data collection was commenced after getting ethical approval from the Institutional Research and Ethical Review Board (IREB) of Khyber Medical College, Peshawar. We used a cross-sectional study design and employed non-probability convenience sampling for collecting data through face-to-face interviews based on the PTSD checklist for DSM-5 (PCL-5). Informed consent was taken from all the participants and their anonymity was ensured. The questionnaire took approximately 5 minutes to complete.

Statistical analysis

Statistical Package for the Social Sciences (SPSS) version 20.0 (Armonk, NY: IBM Corp.) and Microsoft Excel (Redmond, WA: Microsoft Corp.) were used to enter and analyze the data. Using the PTSD checklist for DSM-5 (PCL-5), we found the prevalence of PTSD among burn patients of different ages and genders with different socioeconomic backgrounds and presented it in the form of frequencies, tables, and charts for data analysis. We also found the association of PTSD with gender, age group, and socioeconomic status using chi-square test.

## Results

Demographics

Of the 275 burn patients who participated in our study, 148 (53.8%) were male and 127 (46.2%) were female. The mean age of the patients was 32.48 years. The majority of them were middle-aged adults (n=133, 48.4%). Most of the burn patients in our study belonged to the upper lower class (n=103, 37.5%), whereas the rest belonged to the upper middle, lower middle, and lower classes, respectively. 

Prevalence of PTSD among adult burn patients

**Figure 1:**
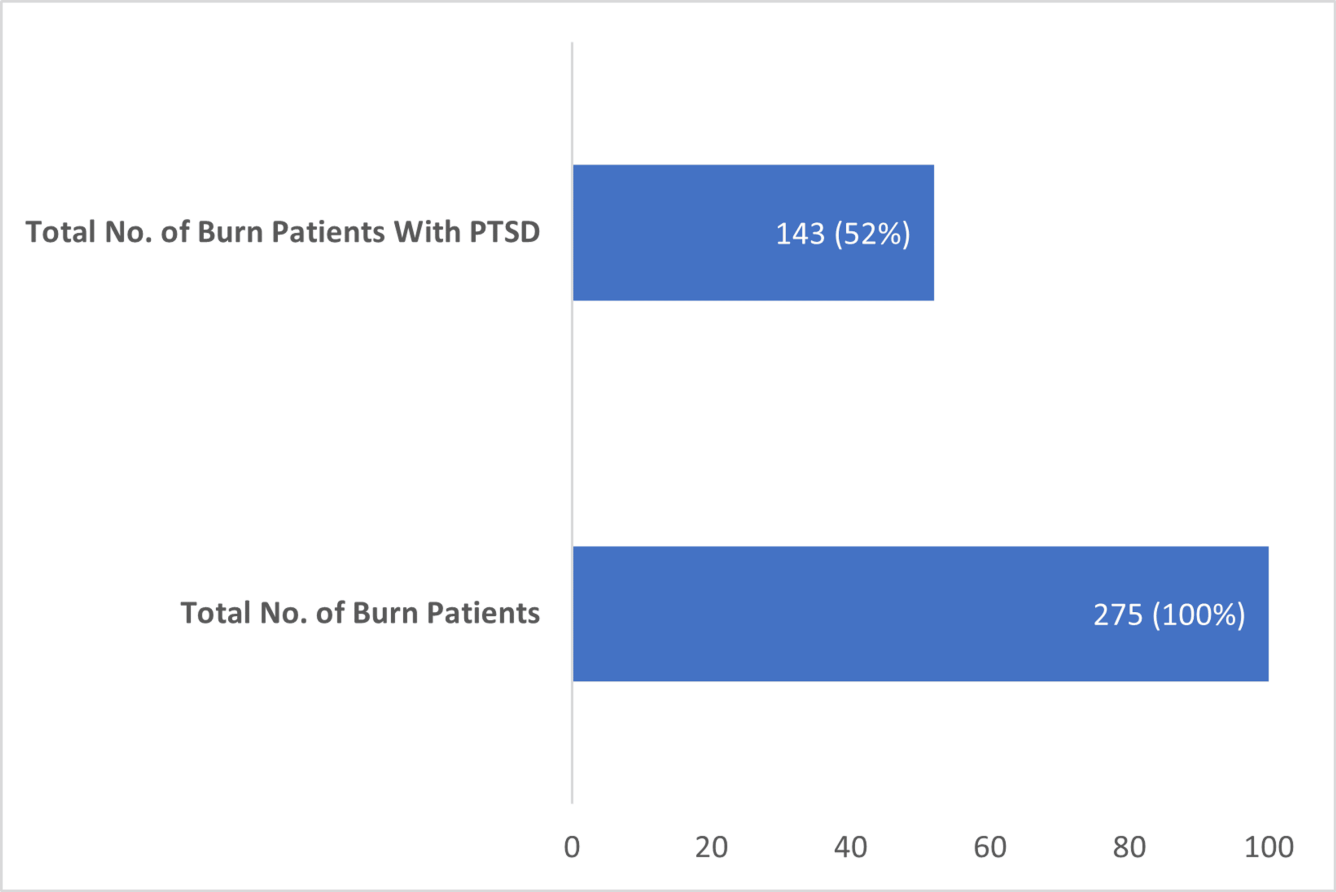
Prevalence of post-traumatic stress disorder. PTSD: post-traumatic stress disorder

The prevalence of PTSD among adult burn patients was investigated using the PTSD checklist for DSM-5. Of the 275 burn patients in our study, we made a provisional diagnosis of PTSD in 143 (52%) burn patients. So, the prevalence of PTSD among adult burn patients in tertiary care hospitals of Peshawar comes out to be 52.0% (Figure 1).

Gender and PTSD

**Figure 2:**
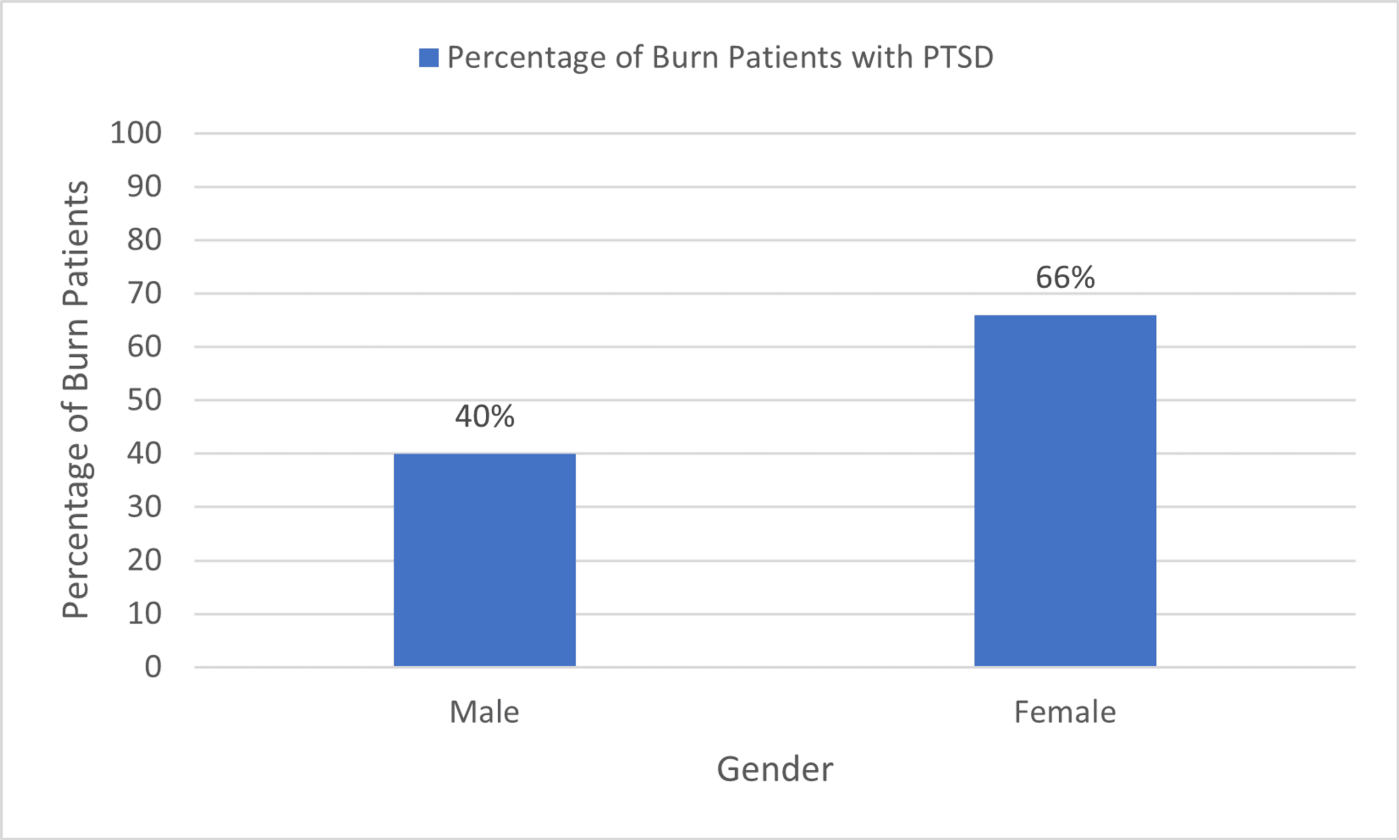
Gender and post-traumatic stress disorder. PTSD: post-traumatic stress disorder

Of the 148 male burn patients in our study, PTSD was provisionally diagnosed in 59 (40%) of them, whereas the prevalence of PTSD in females was 66%, i.e., 84 females were affected out of a total of 127. There was also a statistically significant association between gender and PTSD (p=0.000). These results are shown in Figure 2.

Age groups and PTSD

**Figure 3:**
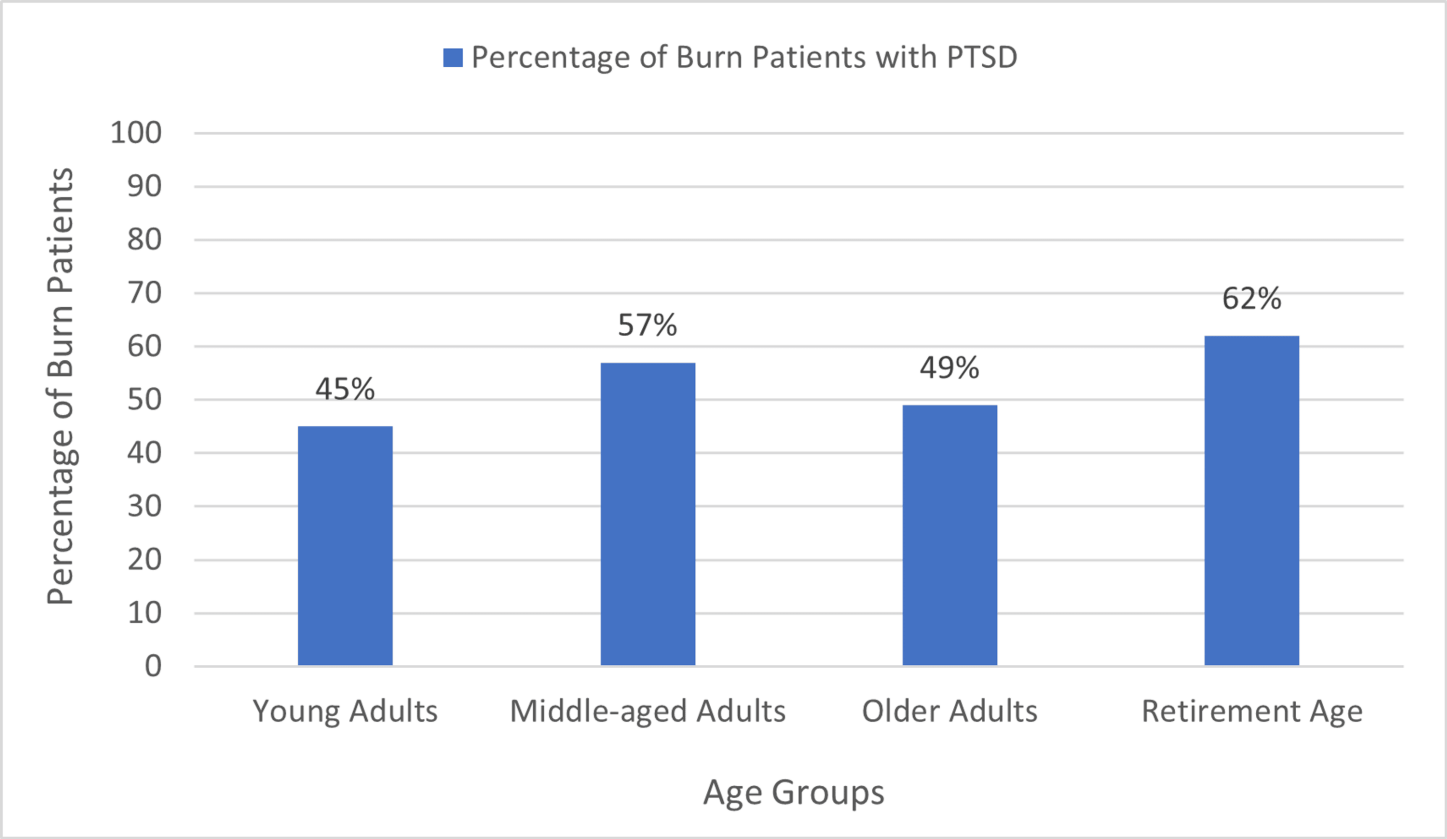
Age groups and post-traumatic stress disorder. PTSD: post-traumatic stress disorder

PTSD was found to be more prevalent in retirement-age patients, with 62% affected (eight out of 13), and in middle-aged adults, with 57% affected (76 out of 133). The association between age groups and PTSD was found to be statistically insignificant (p=0.250). The prevalence of PTSD in different age groups is shown in Figure 3.

Socioeconomic status and PTSD

**Figure 4:**
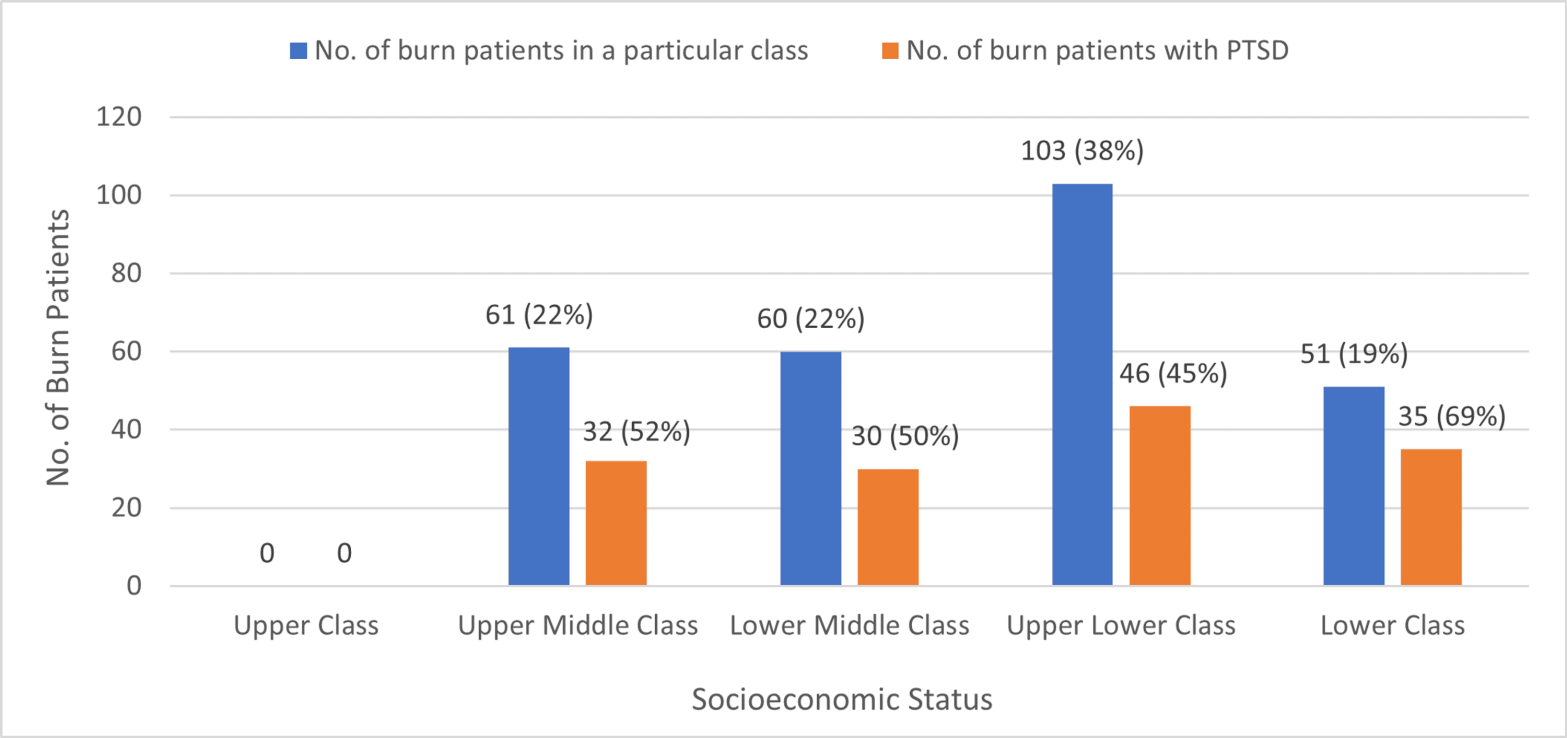
Socioeconomic status and post-traumatic stress disorder. PTSD: post-traumatic stress disorder

The socioeconomic classes were determined by using a modified form of the Kuppuswamy Socioeconomic Status Scale and categorized the burn patients in our study into upper, upper middle, lower middle, upper lower, and lower classes, respectively. PTSD was found to be the most prevalent in the lower class, with nearly 69% of them being afflicted with PTSD, i.e., 35 patients were provisionally diagnosed with PTSD out of a total of 51 lower-class patients. A statistically significant association was found between socioeconomic status and PTSD (p=0.047) (Figure 4).

## Discussion

One of the main findings of our study is that PTSD is very common in burn survivors, which is in agreement with other studies that also found a high prevalence of PTSD in these patients. A Taiwanese study found that 24.8% of burn patients suffered from PTSD three months after the burn accident [22]. Another study found a slightly higher percentage of PTSD in burn survivors, i.e., 38.1% [23]. A study conducted in the United States determined that 24.0% of patients with burn injuries suffered from either post-traumatic stress disorder (PTSD) or acute stress disorder (ASD) [24]. Another Iranian study found the prevalence of PTSD to be 31.5% three months after a traumatic burn injury [25]. The slightly higher prevalence of PTSD in our study could be attributed to several factors including the lack of psychological and social support, the lack of resources and rehabilitation centers, and the lack of post-burn care for burn patients in a developing country like Pakistan. The lack of support from the government to improve the quality of life of these patients and their families is also an important contributing factor to the high prevalence of mental health issues in these burn patients.

We also found that a greater percentage of females were afflicted with PTSD as compared to males. A similar result was found in another study done in Pakistan, which concluded that females had more severe symptoms of PTSD as well as less resilience as compared to males [16]. Another study also found that PTSD as well as other disorders, such as major depression and anxiety, are more prevalent in women than in men [26]. A Danish study found that the prevalence of PTSD in women was two-fold as compared to men [27]. An American Psychological Association study found that although males have a greater risk for trauma, females are more likely to develop PTSD as compared to males. A Nigerian multi-national study also came to the same conclusion [28]. A possible explanation for the greater prevalence of PTSD in females might be the lack of social support and the high incidence of domestic abuse against women in Pakistani society. Most women in Pakistan suffer burns because of domestic violence and interpersonal conflicts, and very little is done to protect these vulnerable women. The higher susceptibility of females to PTSD, the lesser resilience, and the higher incidence of domestic violence-related burn injuries might explain the higher incidence of PTSD in females in our study.

In our study, we found that a greater percentage of middle-aged adults (24-44 years old) and retirement-age (65 years and above) patients suffered from PTSD. Although there is no concrete evidence of a relationship between age and PTSD; however, according to a study done in the United States, young age was associated with more severe symptoms of PTSD [29]. Moreover, the relationship between age and PTSD is still up for debate. The higher incidence of PTSD among burn patients in Pakistan who are middle-aged or retired could be caused by a number of psychological and sociocultural factors. Having a lot of family and financial obligations after a traumatic event can make middle-aged adults more stressed and susceptible to PTSD. In contrast, older adults may have pre-existing medical conditions, less access to mental health care, and a lack of social support, all of which can exacerbate their symptoms. Further complicating age-related trends in PTSD prevalence are cultural stigmas associated with mental health in Pakistan, which may cause younger people to underreport PTSD. These variables underline the need for more investigation to fully comprehend how age and PTSD relate to burn patients in this context.

Another finding of our study was the higher prevalence of PTSD in lower-class patients as compared to upper-middle and lower-middle-class patients. This might be attributed to the fact that lower-income patients have very little psychosocial support and poor post-burn care. Lower-income individuals have poorer access to physical and mental healthcare [30]. They are also at greater risk for trauma, which may contribute to the higher prevalence of PTSD in individuals from lower socioeconomic backgrounds [31]. Another UK study also determined that individuals from lower socioeconomic backgrounds were more vulnerable to trauma as compared to individuals with higher socioeconomic status. Hence, the lack of social and psychological support, substandard access to healthcare, and greater likelihood of trauma among impoverished communities might explain the higher incidence of PTSD in lower socioeconomic class burn patients.

Our study was limited to one major city; hence, more research needs to be conducted to bridge the knowledge gap that exists in Pakistan regarding this topic. This is a cross-sectional study; hence, it neither sheds light on the risk factors that led to the incidence of PTSD nor does it follow the effectiveness of certain coping mechanisms and rehabilitation techniques that might help in mitigating the symptoms of PTSD. There was no follow-up in our study; therefore, more comprehensive longitudinal research, both prospective and retrospective, should be conducted in this area. The use of non-probability convenience sampling limits the generalizability of our findings to the broader burn patient population. Being a cross-sectional study, our research may have recall, selection, and interviewer bias. To reduce these, we used a standardized PTSD checklist and recruited patients from multiple tertiary care hospitals for better representation, and trained interviewers to follow a neutral and standardized approach. These were the limitations of our study. More research needs to be done to understand the relationship between age, gender, socioeconomic status, and PTSD. Further research is required to understand the psychosocial aspect of PTSD in burn patients. The alarmingly high prevalence of PTSD in our study calls for thorough research on post-burn care and devising proper rehabilitation techniques for patients with burn injuries.

Addressing the mental health needs of burn patients requires a multidisciplinary approach because PTSD is very common among them. Early psychological evaluation and prompt intervention can be ensured by incorporating psychology and psychiatry consultations into burn care. Throughout the healing process, a collaborative care model that includes plastic surgeons, mental health specialists, and rehabilitation experts can offer all-encompassing support. Furthermore, organized support services like peer support groups, family education, and counseling can improve social reintegration and resilience. To guarantee that psychological care becomes a crucial component of post-burn treatment and rehabilitation, policymakers should concentrate on developing specialized mental health services within burn units.

## Conclusions

This study aimed to determine the prevalence of PTSD among adult burn patients in tertiary care hospitals, considering variations across different age groups, genders, and socioeconomic statuses. Our study revealed a significantly high prevalence of PTSD among adult burn patients, highlighting that mental health disorders, including PTSD, are often under-recognized and under-treated in burn patients. Additionally, this study showed that burn patients from lower socioeconomic backgrounds, those in retirement age and middle age groups, and female patients were more likely to experience PTSD. The higher prevalence among females may be attributed to their increased vulnerability to mental health conditions such as anxiety, depression, and PTSD. Similarly, individuals from lower socioeconomic backgrounds were more affected due to their limited access to quality healthcare and less awareness of mental health issues. The cross-sectional design and single-city focus of our study restrict our ability to understand long-term recovery and PTSD risk factors. To investigate its psychosocial components and create successful rehabilitation plans, further long-term studies are required.

## Appendices

Questionnaire

We appreciate your willingness to participate in our survey. Your responses will remain anonymous and confidential, and your personal information will not be shared with any third parties. The survey should take approximately 5 minutes to complete. Please answer the questions honestly and to the best of your knowledge, as your feedback will play a crucial role in shaping our research outcomes.

Appendix 1

**Table 2:** Demographic data of participants.

Demographic details
Age: _________
Gender: __________

Appendix 2

**Table 3:** Kuppuswamy Socioeconomic Status Scale. This table presents the Kuppuswamy Socioeconomic Status Scale. It is an open-access tool and has been reformatted into a tabular form for clarity [32,33].

Variables	Responses
(1) Education of head of the family	
(a) Professional degree	
(b) Graduate or postgraduate	
(c) Intermediate or post-high school diploma	
(d) High school certificate	
(e) Middle school certificate	
(f) Primary school certificate	
(g) Illiterate	
(2) Occupation of head of the family	
(a) Professional	
(b) Semi-professional	
(c) Clerical, shop owner, farmer	
(d) Skilled worker	
(e) Semi-skilled worker	
(f) Unskilled worker	
(g) Unemployed	
(3) Monthly income of family (in Pakistani rupees)	
(a) >397236	
(b) 198468-397235	
(c) 149079-198467	
(d) 99384-149078	
(e) 59694-99383	
(f) 20001-59693	
(g) <20000	

Appendix 3

**Table 4:** PTSD checklist for DSM-5 (PCL-5). This table presents the PTSD checklist for DSM-5 (PCL-5) developed by the U.S. Department of Veterans Affairs (VA). PCL-5 is publicly available for clinical and research use from the VA National Center for PTSD. It has been reformatted into a tabular form for clarity [34]. PTSD: post-traumatic stress disorder; DSM-5: Diagnostic and Statistical Manual of Mental Disorders, Fifth Edition; PCL-5: PTSD Checklist for DSM-5

Instructions: Below is a list of problems that people sometimes have in response to a very stressful experience. Keeping your worst event in mind, please read each problem carefully and then select one of the numbers to the right to indicate how much you have been bothered by that problem in the past month.					
In the past month, how much were you bothered by:	Not at all	A little bit	Moderately	Quite a bit	Extremely
1. Repeated, disturbing, and unwanted memories of the stressful experience?	0	1	2	3	4
2. Repeated, disturbing dreams of the stressful experience?	0	1	2	3	4
3. Suddenly feeling or acting as if the stressful experience were actually happening again (as if you were actually back there reliving it)?	0	1	2	3	4
4. Feeling very upset when something reminded you of the stressful experience?	0	1	2	3	4
5. Having strong physical reactions when something reminded you of the stressful experience (for example, heart pounding, trouble breathing, sweating)?	0	1	2	3	4
6. Avoiding memories, thoughts, or feelings related to the stressful experience?	0	1	2	3	4
7. Avoiding external reminders of the stressful experience (for example, people, places, conversations, activities, objects, or situations)?	0	1	2	3	4
8. Trouble remembering important parts of the stressful experience?	0	1	2	3	4
9. Having strong negative beliefs about yourself, other people, or the world (for example, having thoughts such as: I am bad, there is something seriously wrong with me, no one can be trusted, the world is completely dangerous)?	0	1	2	3	4
10. Blaming yourself or someone else for the stressful experience or what happened after it?	0	1	2	3	4
11. Having strong negative feelings such as fear, horror, anger, guilt, or shame?	0	1	2	3	4
12. Loss of interest in activities that you used to enjoy?	0	1	2	3	4
13. Feeling distant or cut off from other people?	0	1	2	3	4
14. Trouble experiencing positive feelings (for example, being unable to feel happiness or have loving feelings for people close to you)?	0	1	2	3	4
15. Irritable behavior, angry outbursts, or acting aggressively?	0	1	2	3	4
16. Taking too many risks or doing things that could cause you harm?	0	1	2	3	4
17. Being “superalert” or watchful or on guard?	0	1	2	3	4
18. Feeling jumpy or easily startled?	0	1	2	3	4
19. Having difficulty concentrating?	0	1	2	3	4
20. Trouble falling or staying asleep?	0	1	2	3	4
